# Supplementary material for: Patterning and dynamics of membrane adhesion under hydraulic stress
Source: Nat Commun. 2023 Nov 17;14:7445. doi: 10.1038/s41467-023-43246-7 (PMC10656516; doi:10.1038/s41467-023-43246-7)
Supplement: Supplementary file 1 — Supplementary information [file 41467_2023_43246_MOESM1_ESM.pdf]

Supplementary Information:
Patterning and dynamics of membrane adhesion under hydraulic stress

Céline Dinet[1,†], Alejandro Torres-Sánchez[2,3,††], Roberta Lanfranco[4], Lorenzo Di Michele[5,6], Marino Arroyo[*2,3,7], and Margarita Staykova[*1]

[1]*Department of Physics, Durham University, Durham, UK*
[2]*Universitat Politècnica de Catalunya-BarcelonaTech, 08034 Barcelona, Spain*
[3]*Institute for Bioengineering of Catalonia (IBEC), The Barcelona Institute of Science and Technology, 08028 Barcelona, Spain*
[4]*Department of Physics, Cavendish Laboratory, University of Cambridge, UK*
[5]*Department of Chemical Engineering and Biotechnology, University of Cambridge, UK*
[6]*Department of Chemistry, Imperial College of London, UK*
[7]*Centre Internacional de Mètodes Numèrics en Enginyeria (CIMNE), 08034 Barcelona, Spain*
[†]*Current affiliation: Laboratoire de Chimie Bactérienne, Institut de Microbiologie de la Méditerranée, CNRS-Aix-Marseille University, 31 Chemin Joseph Aiguier, 13009 Marseille, France*
[††]*Current affiliation: European Molecular Biology Laboratory (EMBL-Barcelona), 08003 Barcelona, Spain*

These authors contributed equally: Céline Dinet, Alejandro Torres-Sánchez.
These authors jointly supervised this work: Marino Arroyo, Margarita Staykova.
Email: marino.arroyo@upc.edu, margarita.staykova@durham.ac.uk.

Contents

Supplementary Figures **3**

Supplementary Table **10**

Supplementary Note 1: Measurement of bond enrichment and mobility **10**

Supplementary Note 2: Theoretical model **12**

Supplementary Note 3: Selection of model parameters **18**

Supplementary References **21**

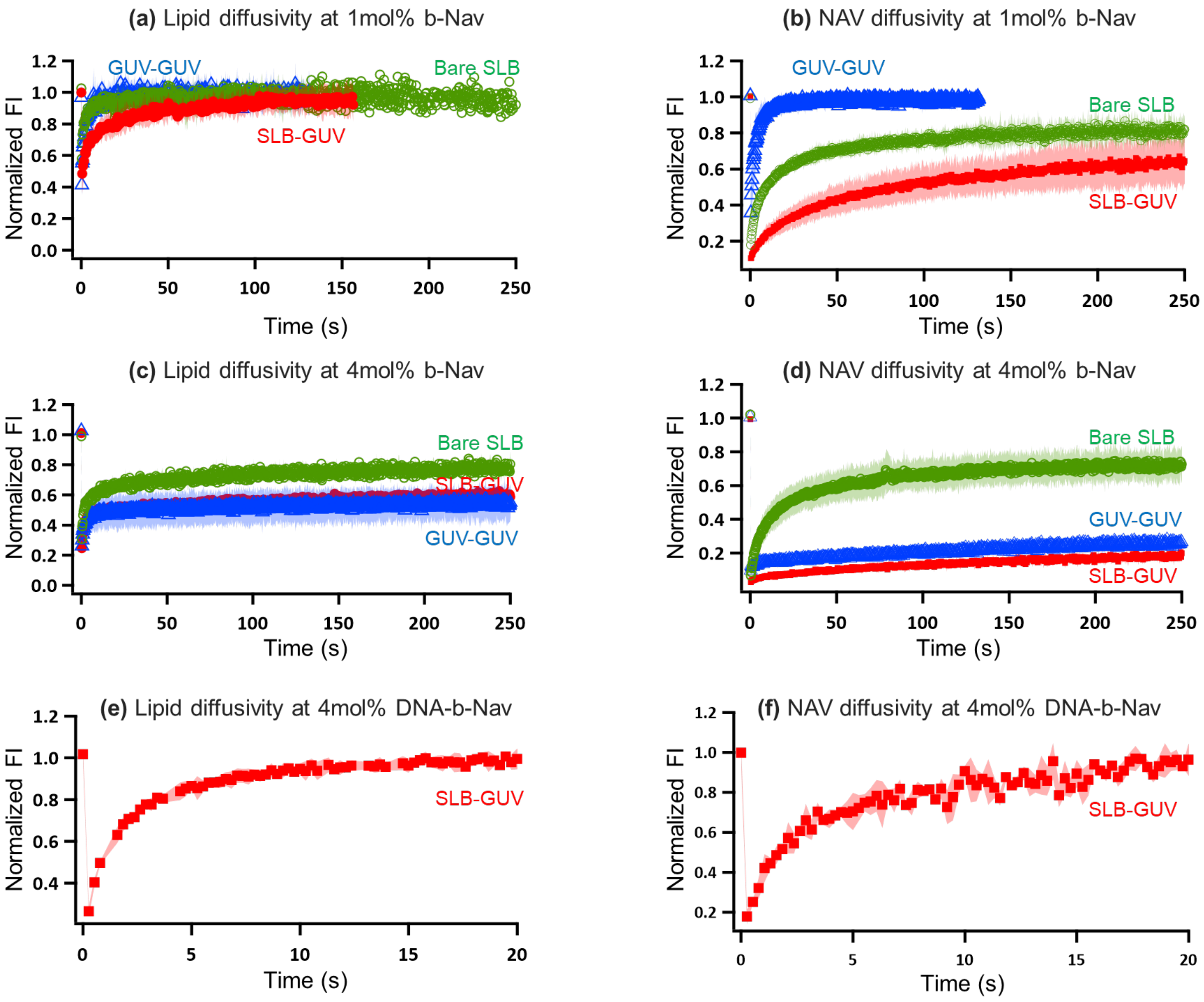

Supplementary Figure 1: Lipids and bond mobilities quantified by Fluorescence recovery after photobleaching (FRAP). Plots show the fluorescent intensity (FI) of the bleached sport over time; FI is normalised to the initial intensity before bleaching and corrected for photo-bleaching during with imaging. (a,b) FI curves for Rhodamine lipids and NAVs respectively at the GUV-SLB (red), GUV-GUV (blue) and NAV- SLB (green) interfaces for systems with 1 mol% biotinylated lipids. (c,d) show the same curves for systems with 4 mol% biotinylated lipids. (e,f) FRAP curves for DNA systems at 4 mol%. All plots show the averaged FI over at least 3 different experiments and the standard deviation.

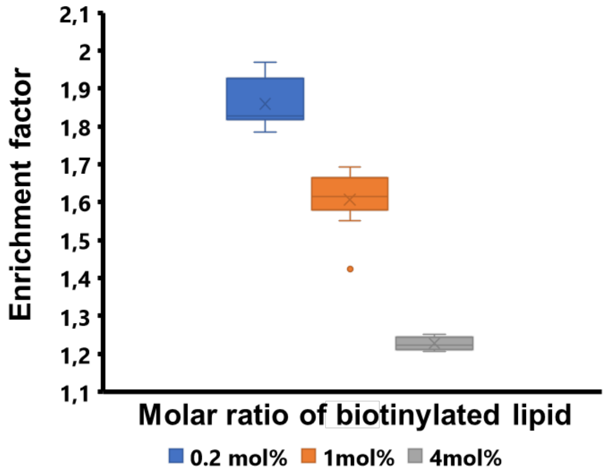

Supplementary Figure 2: Box and whiskers plot of the b-NAV bonds enrichment factor, obtained as the ratio of the mean NAV fluorescent intensity in and outside the adhesion patch in experimental systems with 0.2, 1 and 4 mol% b-DOPE. Data for each condition is obtained from at least 6 repeat measurements obtained from 3 independent experiments.

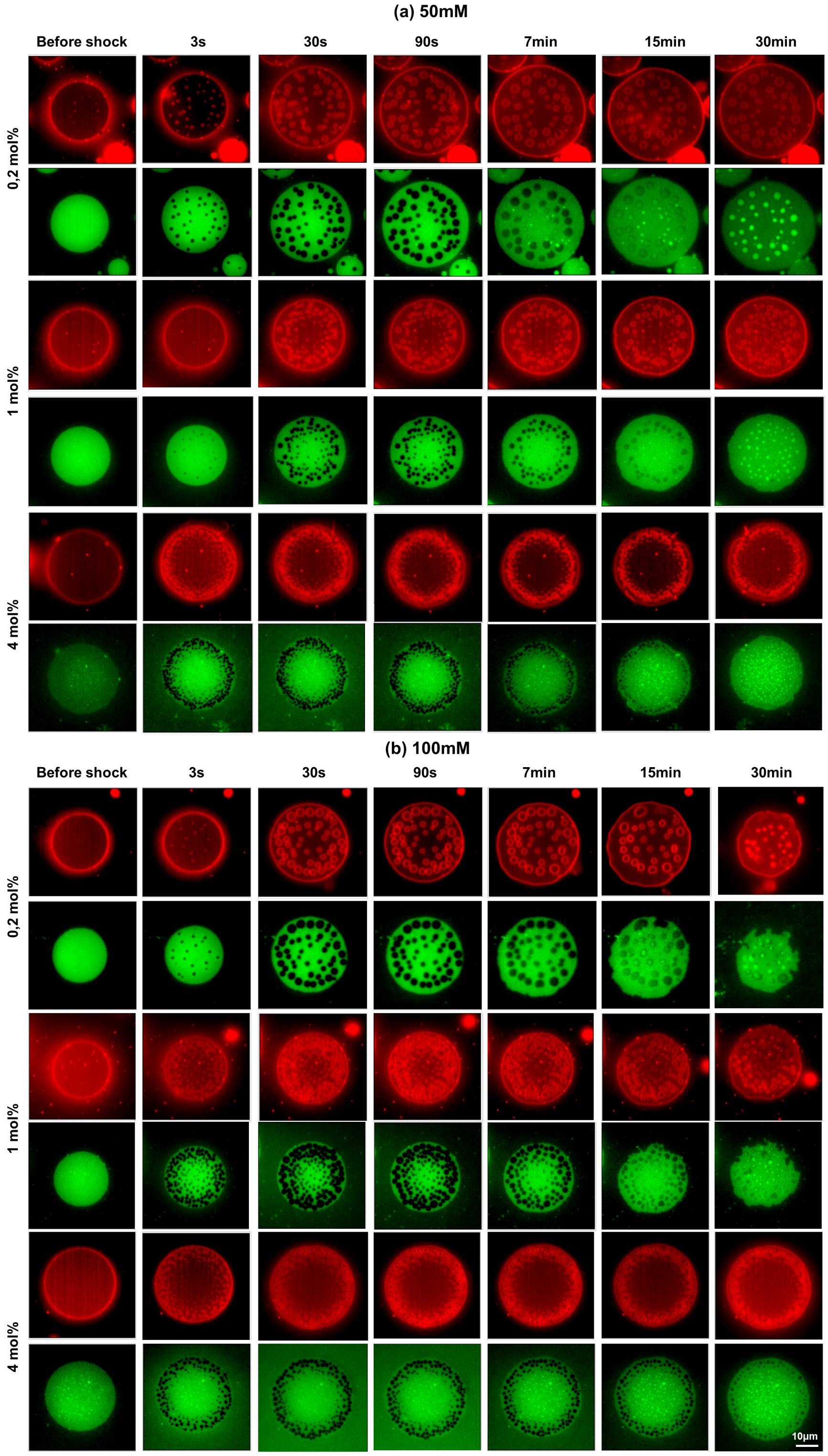

Supplementary Figure 3: Hydraulic fracturing and evolution of blisters in GUV-SLB adhesion patches for osmotic shocks of 50 mOsm (a) and 100 mOsm (b) at linker densities of 0.2, 1 and 4 mol% b-DOPE. The membranes are labeled by Rhodamine-DPPE and appear in red; the NAV is labelled by DyLight488 and appears in green in the images.

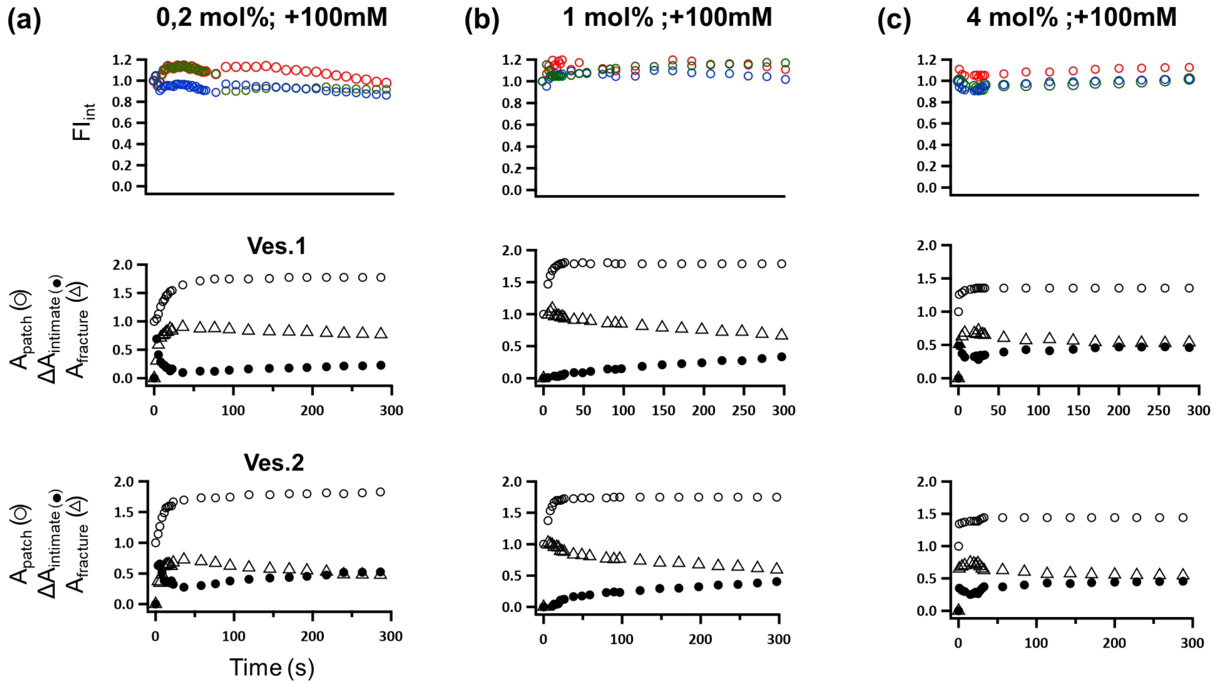

Supplementary Figure 4: Changes in the NAV fluorescent intensity and the adhesion area over time for GUV-SLB systems with 0.2 (a), 1 (b) and 4 mol% (c) biotinylated lipids. The NAV fluorescent intensity is integrated over the area of intimate membrane adhesion (FI_{int}). Data for FI_{int} is obtained from three vesicles from 2 independent experiments. Furthermore, for each bond density we show two different vesicle data sets analysed for: Total adhesion patch area, normalised to the initial area (A_{patch}); Total fracture area (A_{fracture}) and change in intimate adhesion area ($\Delta A_{\mathrm{intimate}}$) over time, both as a fraction of the total patch area spreading, $A_{\mathrm{patch}} - A_{\mathrm{patch}}(0)$. The fracture area A_{fracture} is measured when it is maximum.

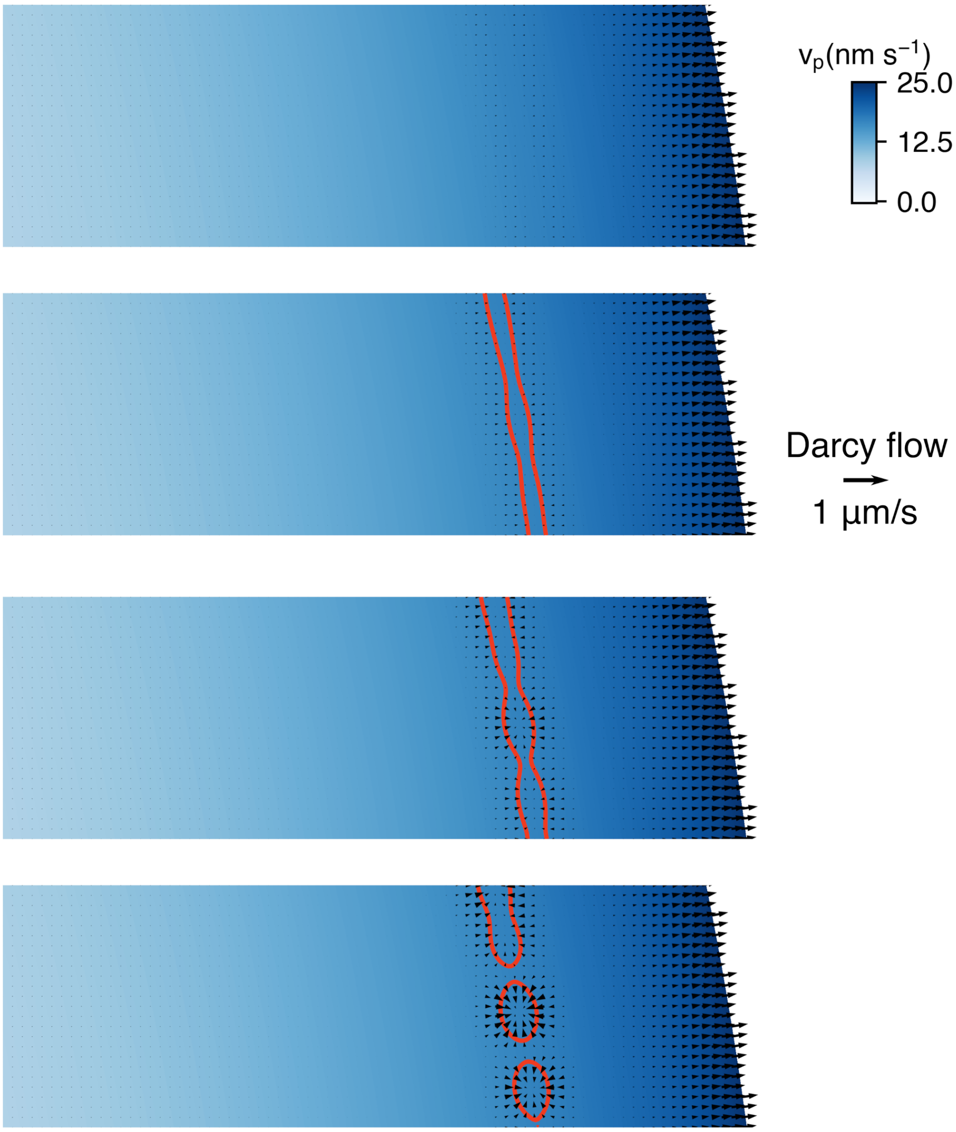

Supplementary Figure 5: Detail of symmetry-breaking instability by which an annular blister fragments into blisters with circular footprint. The color map is permeation velocity and the arrows lateral Darcy flow. The outline of the blister is marked in red.

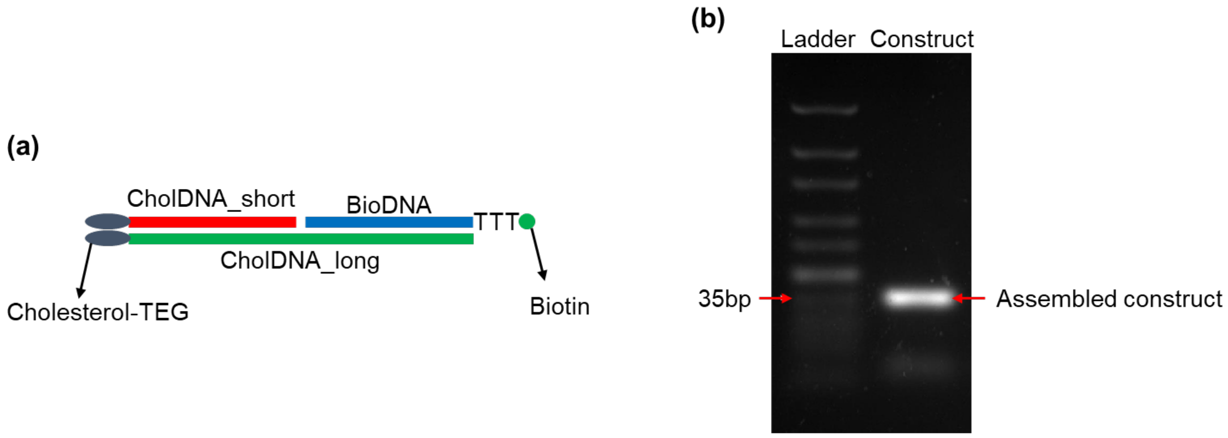

Supplementary Figure 6: Structure and characterisation of biotinylated, double-cholesterol DNA linkers. (a) Schematics of the DNA linkers and their single-stranded components. Oligonucleotide sequences are given in the methods section. (b) Agarose gel electrophoresis shows correct assembly of the constructs, which form a sharp band aligned with the 35bp band of the ladder (Ultra Low Range DNA ladder, Invitrogen). A faint secondary band at lower molecular weight is the result of a slight deviation from stochiometric oligonucleotide concentration arising from pipetting uncertainties. Non-cholesterolised versions of the CholDNA_Short and CholDNA_Long strands were used to assembled the constructs for agarose experiments, to prevent micellisation.

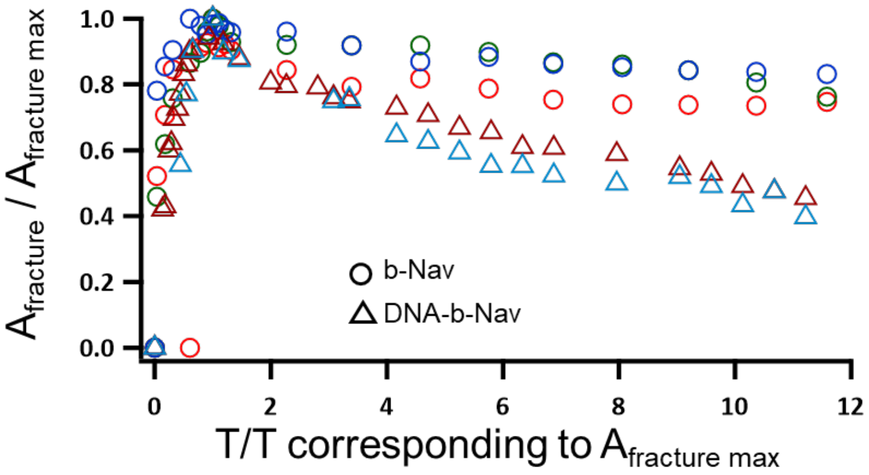

Supplementary Figure 7: Comparison between the changes in $A_{fracture}$ as a function of time for DNA-b-NAV and for b-NAV samples. A_{fracture} has been normalised to the maximum fractured area ($A_{\mathrm{fracture}}/A_{\mathrm{fracture\ max}}$) and time is normalized to the time when A_{fracture} is at maximum ($t/t_{@A_{\mathrm{fracture\ max}}}$). The data from b-NAV samples (circles) is obtained from 3 different vesicles at 4 mol% biotin density and subject to 100 mM osmotic shock taken from two independent experiments. The DNA-b-NAV data (triangles) is taken from 2 vesicles both subject to 100 mM osmotic shock, one prepared at 4 mol% biotin density and the other at 6 mol%. The DNA samples shows much quicker decrease in A_{fracture}, even at higher bond density, which is a signature of enhanced Ostwald ripening and quicker discharge of interstitial water.

Parameter	Variable	Units	Value
Initial bond tension	γ_0	kPa µm (= mN m^{-1})	$2.5 \cdot 10^{-2}$
Initial vesicle radius	R_0	µm	18.75
Initial vesicle contact angle	θ_0	deg	127
Initial osmotic pressure	Π_e^0	kPa	750
Bond resting length	z_0	µm	$7 \cdot 10^{-3}$
Bond compliance length	ℓ_k	µm	$7 \cdot 10^{-3}$
Osmotic shock	$\delta\Pi$	kPa	125
Osmolyte diffusivity	D	µm^2 s^{-1}	50
Reference water mobility	α_0	µm^2 s^{-1} kPa^{-1}	0.1
Reference membrane friction	μ_0	kPa s µm^{-1}	0.1
Membrane permeability	K	µm s^{-1} kPa^{-1}	$2 \cdot 10^{-4}$
Membrane viscosity	η	kPa µm s	10^{-5}

Supplementary Table 1: Parameters used for the simulation in Fig. 2 and Video 2. Changes to these parameters in other figures or videos are explicitly mentioned in the corresponding captions. The data characterizing the initial state prior to the shock (first three lines) can be easily mapped to the initial data given in Box 1 (number of bonds N_b, number of trapped osmolytes N_o, vesicle surface area S). The bond compliance length measures is the amplitude of typical bond thermal fluctuations and also the critical separation distance for loss of stability of an adhesion. It is given by $\ell_k = \sqrt{k_B T/k}$. The osmotic shock $\delta\Pi = 125$ kPa corresponds to 50 mM.

Supplementary Note 1: Characterisation of experimental systems

Geometry of adhered vesicles

Most of the vesicles in our experiments have a diameter between 20-30 µm. When adhered to a supported bilayer they have the shape of a truncated sphere with height (h) and basal radius (r) (main text, Fig. 2). In equilibrium before the osmotic shock, the vesicles have a contact angle of about 120 degrees, which decreases to about 90 degrees after the shock. The volume loss of the vesicle can be estimated by measuring h and r from the XZ cross-sectional vesicle images, and by using the relation $V = \pi h(3r^2 + h^2)/6$, where V is the volume of the spherical cap. In Figure 1a, h decreases from 19 to 14 µm and r increases from 11.5 to 15 µm before and after the osmotic shock, which corresponds to 20% volume decrease in the vesicle.

Bond density

In the experimental systems that use biotin- neutravidin (b-NAV) to link the two membranes, we worked with 0.2, 1 and 4 mol% of b-DOPE in the DOPC lipid mixture. Assuming that DOPC and b-DOPE have a similar surface area of 70×10^{-8} µm^2, then the density of biotinylated lipids in the three types of vesicles is $2.8{\times}10^3$, $1.4{\times}10^4$ and $5.7{\times}10^4$ µm^{-2}, respectively. In its lowest energy configuration, the neutravidin linker binds to two biotins from each opposing membrane, hence the NAV densities are half the biotin densities. In addition when membranes with mobile linkers adhere, there is an enrichment of linkers in the adhesion zone, which can be estimated from the ratio of NaV fluorescence intensities from the adhesion zone and outside. For the 0.2, 1 and 4 mol% of biotinylated membranes the enrichment factors are approximately 1.9, 1.6 and 1.2 (Fig. S2). Therefore the NAV densities in the adhesion zone are $\sim 2.7 \times 10^3$, 1.1×10^4 and 3.4×10^4 µm^{-2}, respectively. These densities have been used to calculate the initial osmotic pressure of bonds (see SI section 3). It has been previously estimated that membranes with 5 mol% of b-DOPE correspond to a maximum packing of NAV linkers in the interstitial space[1]. Therefore our systems with 4 mol% b-DOPE are close to bond saturation.

Experiments with DNA spacers

Our experiments with DNA spacers were aimed at the same bond density as the b-NAV experiments at 1 and 4 mol% b-DOPE. However, the actual density of DNA linkers is likely to be smaller due to the non-specific adhesion of DNA to PDMS and glass surfaces, as well as strong steric/Coloumb repulsion between the DNA constructs in the membrane[2]. To compensate for this underestimation and achieve the maximum possible DNA density, we added up to 8mol % b-DNA linkers to the membranes.

Thermal mobility of lipids and linkers

In systems with 1 mol% b-DOPE, the thermal mobility of b-NAV bonds and lipids between two vesicles (GUV-GUV) is very high, compared to GUV-SLB systems where the lipid and b-NAV mobility are affected by interactions with substrate (Fig. S3a,b)[1]. Comparing the FRAP curves for GUV-SLB patches to bare SLBs with b-NAV complexes allows us to distinguish between the effects of substrate and membrane-membrane adhesion. It can be seen from Fig. S3b that the substrate can significantly impact NAV mobility and recovery presumably by non-specific interactions and pining[1].

As we increase the biotinylated fraction of lipids to 4 mol% we observe strong impediment of lipid and NAV mobilities (Fig. S3c,d), due to the lateral crowding of the bulky NAV receptors. In fact, it was proposed that the NAVs form a glassy layer above the membrane that affects lipid mobility by friction[1]. Consistent with this the fraction of mobile lipids is reduced by 50%. The recovery of the NAV signal in both GUV-SLB and GUV-GUV systems is reduced by more than 75% and it never reaches a plateau suggesting that NAV diffusion is stalled.

The FRAP experiments on DNA systems show fast and complete lipid and bond recoveries (Fig. S3e,f). The reasons for these high mobilities may be different. On one hand it is likely that the bond density in the

DNA systems does not reach 4 mol% for reasons discussed in the previous section. Second, since the NAVs are removed from the membrane surface, the lipid mobility is not impacted by the NAVs. Finally, the links between the DNA rods and the cholesterol anchors on the membrane end, and the biotin at the other end, are flexible. Hence NAVs can diffuse much easier past each other even at higher NAV density.

Supplementary Note 2: Theoretical model

We develop next a theoretical model for the formation of pockets in the interstitial zone of a vesicle adhered to a supported lipid bilayer after the application of an osmotic shock. We model the vesicle as a spherical cap of basal radius $r(t)$ and height $h(t)$. Therefore, the supported lipid bilayer is a disk $\mathcal{D}(t)$ in the (x, y) plane of radius $r(t)$. The shape of the vesicle in the interstitial zone, $\Gamma(t)$, is characterised by a Monge parametrisation of the form $z(x, y, t)$.

We denote the hydraulic and osmotic pressures in the external medium as P_e and Π_e. Analogously, we define these quantities in the inside of the vesicle P_i and Π_i. In equilibrium, osmolytes cannot cross the semi-permeable membrane and hence sustain an osmotic pressure difference $\Delta\Pi = \Pi_\mathrm{i} - \Pi_\mathrm{e}$ between that of the external medium and the vesicle interior, given by $\Pi_\mathrm{i} = k_\mathrm{B}TN_\mathrm{o}/V$ with N_o the number of trapped osmolytes and V the vesicle volume. Instead, water can equilibrate across the membrane, and hence its chemical potential inside, which is proportional to $P_\mathrm{i} - \Pi_\mathrm{i}$, is equal to that outside, leading to $\Delta\Pi = \Delta P$.

Transport of osmolytes and water in the interstitial region

In the interstitial space, we consider the concentration of osmolytes $n(x, y, z, t)$ and the mechanical pressure $P(x, y, z, t)$. Given that the thickness of the interstitial zone is small (of a few nanometers compared to the size of the vesicle of $\sim 10-20$ µm), we consider that $n(x, y, z, t) = n(x, y, t)$ and $P(x, y, z, t) = P(x, y, t)$. Given that the interstitial region is crowded with the linkers joining the supported lipid bilayer and the vesicle, we consider the interstitial region to be a porous medium of mobility α. Then, lateral fluid flows in the interstice are characterised by the Darcy law

$$\mathbf{v}^{\text{fluid}} = -\alpha\nabla P. \tag{1}$$

Water can cross the membrane with a permeability K, which leads to a permeation rate proportional to the difference of water chemical potential given by

$$v_\mathrm{p} = K[(P - \Pi) - (P_\mathrm{i} - \Pi_\mathrm{i})], \tag{2}$$

Neglecting the volume of adhesion molecules in the dilute limit, incompressibility of the fluid beneath the monolayer can be expressed as

$$\partial_t z + \nabla\cdot\left(z\mathbf{v}^{\text{fluid}}\right) - v_\mathrm{p} = \partial_t z - \nabla\cdot(z\alpha\nabla P) - K[(P - \Pi) - (P_\mathrm{i} - \Pi_\mathrm{i})]j = 0, \tag{3}$$

where $j(x, y, t) = \sqrt{1 + |\nabla z(x, y, t)|^2}$ is the ratio between the differential of surface and its projection onto the $xy-$plane at a given point (x, y) and time t, i.e. $j(x, y, t)\, dxdy = dS$. Osmolytes are advected by the fluid and they can also diffuse with a diffusivity D following Fick's law. Their net velocity is then

$$\mathbf{v}^{\text{osmolytes}} = \mathbf{v}^{\text{fluid}} - D\frac{\nabla n}{n}. \tag{4}$$

Since the bilayer is impermeable to osmolytes, conservation of number of osmolytes beneath the monolayer leads to

$$\partial_t(zn) + \nabla\cdot\left(zn\mathbf{v}^{\text{osmolytes}}\right) = \partial_t(zn) - \nabla\cdot[z(\alpha n\nabla P + D\nabla n)] = 0. \tag{5}$$

We can rewrite this equation directly in terms of the osmotic pressure by multiplying the previous equation by $k_\mathrm{B}T$ and assuming a van't Hoff relation $\Pi = k_\mathrm{B}Tn$ to obtain

$$\partial_t(z\Pi) - \nabla\cdot[z(\alpha\Pi\nabla P + D\nabla\Pi)] = 0. \tag{6}$$

Finally, the boundary conditions for Π and P are simply

$$P = P_\mathrm{e}, \qquad \text{at } \partial\mathcal{D}, \tag{7}$$

$$\Pi = \Pi_\mathrm{e}, \qquad \text{at } \partial\mathcal{D}, \tag{8}$$

since the interstice can exchange fluid and osmolytes with its surroundings and we assume that the external pressures are unaltered during the dynamics.

Membrane mechanics in the adhesion patch

In the absence of bending moments, which we neglect for simplicity, balance of forces tangential and normal to the surface can be expressed as

$$\nabla_S \cdot \boldsymbol{\sigma} + \mathbf{b} = \mathbf{0}, \tag{9}$$

$$\boldsymbol{\sigma} : \mathbf{k} + b_n = 0. \tag{10}$$

where $\boldsymbol{\sigma}$ is the stress tensor of the membrane (with units of tension) and $\mathbf{b}$ and b_n are the tangential and normal components of the external force density, i.e. $\mathbf{B} = \mathbf{b} + b_n\mathbf{N}$ is the three-dimensional body force per unit area where $\mathbf{N}$ is the normal to the membrane, which is given by $\mathbf{N} = (\mathbf{e}_1 \times \mathbf{e}_2)/|\mathbf{e}_1 \times \mathbf{e}_2|$ with $\mathbf{e}_i$ the basis of the tangent space to Γ given by the Monge parametrization, $\mathbf{e}_1 = (1, 0, \partial_x z)$, $\mathbf{e}_2 = (0, 1, \partial_y z)$. We can obtain the tangential component using the projector $\mathbf{P} = \mathbf{I} - \mathbf{N} \otimes \mathbf{N} = \mathbf{e}^i \otimes \mathbf{e}_i$, with $\mathbf{e}^i$ the dual basis to $\mathbf{e}_i$, as $\mathbf{b} = \mathbf{P} \cdot \mathbf{B}$. Here ∇_S denotes the surface covariant derivative. We model the stress tensor of the membrane as

$$\boldsymbol{\sigma} = (\sigma - \gamma)\mathbf{I} + \boldsymbol{\sigma}_v, \tag{11}$$

where σ is the bare surface tension (the partial tension of lipids), $-\gamma$ is the osmotic tension generated by the bonds and

$$\boldsymbol{\sigma}_v = 2\eta \left[\mathbf{d} - \frac{1}{2}(\mathrm{tr}\mathbf{d})\mathbf{I} \right], \tag{12}$$

is the viscous stress with η the membrane viscosity. The rate-of-deformation tensor of the surface takes the form

$$\mathbf{d} = \frac{1}{2}\left[\nabla_S \mathbf{v} + (\nabla_S \mathbf{v})^T \right] - v_n \mathbf{k}, \tag{13}$$

where $\mathbf{k}$ is the curvature tensor given by $k_{ab} = \mathbf{N} \cdot \partial_b \mathbf{e}_a$, and $\mathbf{v}$ and v_n the tangential and normal velocities of the membrane. In a dilute approximation, the osmotic 2D pressure of bonds can be expressed as $\gamma = k_B T c$ following a van't Hoff relation with c the number concentration of bonds per unit membrane area.

We consider hydraulic, bond-related, and frictional force densities acting on the membrane as

$$\mathbf{B} = \mathbf{B}_p + \mathbf{B}_b + \mathbf{B}_f, \tag{14}$$

where

$$\mathbf{B}_p = (P - P_i)\mathbf{N} \tag{15}$$

is the force density generated by the difference of mechanical pressure across the vesicle,

$$\mathbf{B}_b = -c\mathcal{V}'(z)\mathbf{E}_3, \tag{16}$$

is the force generated by the bonds in the direction perpendicular to the adhesion patch ($\mathbf{E}_3$ is the unit vector in the direction of z), with $\mathcal{V}'(z)$ the force born by a single bond and $\mathcal{V}(z)$ the single bond stretching potential, and

$$\mathbf{B}_f = -\mu \mathbf{v}, \tag{17}$$

is a frictional force with friction coefficient μ tangent to the membrane. We further impose the constraint of membrane inextensibility

$$\mathrm{tr}\,\mathbf{d} = \nabla_S \cdot \mathbf{v} - v_n H = 0, \tag{18}$$

where $H = \mathrm{tr}\,\mathbf{k}$ is the total curvature and σ acts as a Lagrange multiplier. Plugging these expressions in Eqs. (19) and (20), we get

$$\nabla_S(\sigma - \gamma) + 2\eta\nabla_S \cdot \mathbf{d} + \mathbf{P} \cdot \mathbf{B}_b = \mu\mathbf{v}, \tag{19}$$

$$(\sigma - \gamma)H + 2\eta\,\mathbf{d} : \mathbf{k} + P - P_i - T_{\mathrm{bonds}} j^{-1} = 0. \tag{20}$$

where we have defined $T_{\mathrm{bonds}} = c\mathcal{V}'$ and used that $\mathbf{N} \cdot \mathbf{E}_3 = j^{-1}$. These equations along with the boundary condition

$$\sigma = \sigma_v \qquad \text{at } \partial\mathcal{D}, \tag{21}$$

with σ_v the tension on the detached part of the vesicle, can be used to solve for the membrane velocity $\mathbf{v}$, and hence integrate z in time.

Bond dynamics in an adiabatic approximation

We consider that bonds are parallel to the z axis and join the point $(x, y, 0)$ in the supported lipid bilayer with the point (x, y, z) in the vesicle. We then define the number density of bonds attached at a given point of the vesicle in the adhesion patch per unit membrane area $c(x, y, t)$. Assuming that bonds can equilibrate by diffusion infinitely fast, they would reach the Boltzmann distribution given by

$$c(x, y, t) = C(t) \exp\left(-\frac{\mathcal{V}(z(x, y, t))}{k_B T}\right), \tag{22}$$

where C is a normalization constant that can be obtained from the conservation of the number of bonds in the attached area

$$\int_{\Gamma_t} c dS = c_0 A_0 \Rightarrow C = \frac{1}{c_0 A_0} \int_{\Gamma_t} \exp\left(-\frac{\mathcal{V}}{k_B T}\right) dS. \tag{23}$$

The Boltzmann distribution of bonds can be understood by writing the chemical potential of bonds as $\mu = \mu_0 + k_B T \log c + \mathcal{V}(z)$, where μ_0 is the standard chemical potential, the second term is the mixing entropy part and the last term is the stretching part[3;4]. The Boltzmann distribution immediately follows from assuming fast equilibration of the chemical potential of bonds, i.e. that μ is constant in the patch.

Note then that in these equilibrium conditions, one can define an effective potential generated by the bond ensemble as

$$U(z) = -k_B T c(z) = -k_B T C \exp\left(-\frac{\mathcal{V}(z)}{k_B T}\right), \tag{24}$$

which immediately satisfies

$$\gamma = k_B T c = -U, \tag{25}$$

$$\mathbf{B}_b = -c\mathcal{V}'(z)\mathbf{E}_3 = -U'(z)\mathbf{E}_3. \tag{26}$$

Plugging these expressions in Eq (19), one can write the force balance equation tangential to the membrane as

$$\nabla_S \sigma + 2\eta \nabla_S \cdot \mathbf{d} = \mu \mathbf{v}, \tag{27}$$

since $-\nabla_S \gamma + \mathbf{P} \cdot \mathbf{B}_b = \nabla_S U(z) - U'(z)\mathbf{P} \cdot \mathbf{E}_3 = U'(z)\nabla_S z - U'(z)\mathbf{P} \cdot \mathbf{E}_3 = U'(z)(\nabla_S z - \mathbf{P} \cdot \mathbf{E}_3) = U'(z)\left[\nabla_S z - \mathbf{E}^i(\mathbf{E}_i \cdot \mathbf{E}_3)\right] = U'(z)\left[\nabla_S z - \mathbf{E}^i \partial_i z\right] = 0$ since $\nabla_S z = \mathbf{E}^i \partial_i z$. Hence, the osmotic tension of bonds does not affect force balance tangent to the surface.

Mechanics of the vesicle

Assuming the vesicle is a spherical cap of basal radius r and height h and a homogeneous surface tension σ_v, the law of Laplace leads to

$$\frac{2\sigma_v}{R} = P_e - P_i. \tag{28}$$

where $R = (r^2 + h^2)/2h$ is the radius of curvature of the vesicle. Balance of forces at the edge of the adhesion zone is given by an averaged Young-Dupré law

$$\sigma_v \left(1 + \int_{\partial \mathcal{D}(t)} \cos\theta dl\right) = -\frac{1}{2\pi r} \int_{\partial \mathcal{D}(t)} U dl, \tag{29}$$

where $\cos\theta = 1 - (h + (z - z_0))/R$ is the contact angle. Given the volume of the spherical cap $V = \pi h(3r^2 + h^2)/6$ and the volume of the interstitial zone $V^{\text{interstice}} = \int_{\mathcal{D}(t)} z dx dy$, conservation of volume in the vesicle reads

$$\dot{V} - \dot{V}^{\text{interstice}} + \pi(r^2 + h^2) K \left[(P_e - \Pi_e) - (P_i - \Pi_i)\right] + \int_{\Gamma_t} K \left[(P - \Pi) - (P_i - \Pi_i)\right] dS = 0. \tag{30}$$

Substituting $\dot{V} = \pi(r^2 + h^2)\dot{h}/2 + \pi h\dot{r}$ and $\dot{V}^{\text{interstice}} = \int_{\mathcal{D}(t)} \partial_t z dx dy + \dot{a} \int_{\partial\mathcal{D}(t)} z dl$,

$$\pi(r^2 + h^2)\dot{h}/2 + \left(\pi h - \frac{1}{r}\int z dx dy\right)\dot{r} - \int_{\Omega_t} \partial_t z dx dy$$
$$+ \pi(r^2 + h^2)\eta\left[(P_{\text{e}} - \Pi_{\text{e}}) - (P_{\text{i}} - \Pi_{\text{i}})\right] + \int_{\Gamma_t} \eta\left[(P - \Pi) - (P_{\text{i}} - \Pi_{\text{i}})\right] dS = 0. \quad (31)$$

Finally, conservation of membrane area can be written as

$$\pi(r^2 + h^2) + \int_{\Gamma_t} dS = A, \quad (32)$$

where A is the area of the vesicle. This is equivalent to

$$\left[2\pi r + \int_{\partial\mathcal{D}(t)} |\nabla z|^2 dl\right]\dot{r} + \pi h\dot{h} - \int_{\Gamma_t} v_n H dS = 0. \quad (33)$$

Finally, given that the number of osmolytes is fixed in the vesicle,

$$\Pi_{\text{i}} = \Pi_{\text{i}0}\frac{V_0}{V}. \quad (34)$$

Eqs. (28), (29), (31), (33) and (34) allow us to to solve for $\dot{r}$, $\dot{h}$, P_{i}, σ_{v} and Π_{i}, and hence obtain the vesicle shape over time, noting that these equations depend on the patch problem through v_n, $z(x, y, t)$, $P(x, y, t)$ and $\Pi(x, y, t)$.

Initial state

The initial equilibrium state of the adhered vesicle is controlled by the physics of two semi-permeable interfaces of different dimension: the membrane and the adhesion rim. Because water can cross the membrane but osmolytes cannot, the water chemical potential can reach equilibrium whereas osmolytes generate an osmotic pressure difference sustained by membrane tension σ_{v} following Laplace's law. Analogously, lipids can cross the adhesion rim but bonds cannot, leading to an adhesion tension γ, here the 2D osmotic pressure of bonds trapped within the adhesion rim, $\gamma = k_{\text{B}}Tc$[3;5]. This tension is balanced by membrane tension according to a Young-Dupré relation $\gamma = \sigma_{\text{v}}(1 - \cos\theta)$. The osmotic shock drives water efflux and disrupts all these equilibrium conditions. Because the excess osmolytes from the shock can penetrate the interstice, water drains from the vesicle into the cleft, leading to a local swelling of the interstice at a distance from the edge as discussed in the main text.

Driving force for bond motion during FRAP and blister formation

The chemical potential of bonds is given by

$$\mu(c, z) = \mu_0 + k_{\text{B}}T \log c + \mathcal{V}(z), \quad (35)$$

where μ_0 is a reference chemical potential. Gradients of the chemical potential drive bond motion. For a harmonic potential with stiffness k, the stretching term becomes dominant for bond elongations $> \sqrt{k_{\text{B}}T/k} \sim$ 1 nm.

Assuming linear drag of bonds at a microscopic scale, this form of the chemical potential leads to a diffusion equation for the bond distribution biased by bond bond stretching[4]. Instead, a microscopic picture of biased Brownian motion with stick-slip nonlinear friction[6;7] leads to a transport behavior that strongly depends on the magnitude of the driving force.

FRAP only introduces gradients in the entropic term of $\mu(c, z)$ due to the unmixing of bleached and unbleached molecules, which drive fluorescence recovery. Instead, fracture separation occurs at the expense

of the entropic term, since it leads to the generation of a region devoid of bonds, and is driven by the gradients in the stretching term. Driven by strong osmotic effects, these mechanical gradients can be much larger than entropic ones, and result in apparently mobile bonds during the strongly out-of-equilibrium initial phases of blister formation, and partially immobile bonds under FRAP or as the system approaches equilibrium.

Theory Box

The adhesion patch on the SLB is a disk $\mathcal{D}(t)$ in the (x, y) plane of radius $r(t) = R(t)\cos\theta(t)$. The adhesion patch on the neighboring vesicle $\Gamma(t)$ is described by the height function $z(x, y, t)$ with $(x, y) \in \mathcal{D}(t)$. Local areas on $\Gamma(t)$ and $\mathcal{D}(t)$ are related by $dS = j\,dxdy$ where $j = \sqrt{1 + [\nabla z]^2}$. Here $\nabla = (\partial_x, \partial_y)$, whereas the surface nabla operator is ∇_S.

Vesicle unknowns: Radius of spherical cap $R(t)$, contact angle $\theta(t)$, tension $\sigma_v(t)$, osmotic pressure $\Pi_i(t)$ and hydraulic pressure relative to that in the external medium $P_i(t)$.

Unknowns in the adhesion patch: membrane height $z(x, y, t)$; osmotic pressure $\Pi(x, y, t)$ and hydraulic pressure $P(x, y, t)$ in the interstice relative to that in the external medium; tangential velocity $\mathbf{v}(x, y, t)$, bare tension $\sigma(x, y, t)$ and bond number density $c(x, y, t)$ on the membrane $\Gamma(t)$.

Bond distribution in an adiabatic approximation. Assuming bonds redistribute fast compared to osmolytes, water and membrane, and accounting for conservation of their number N_b, the equilibrium Boltzmann distribution is given by

$$c(x, y, t) = C(t)\exp\left(-\frac{\mathcal{V}(z(x, y, t))}{k_B T}\right) \quad \text{subject to} \quad N_b = \int_{\Gamma(t)} c(x, y, t)dS \tag{36}$$

where $\mathcal{V}(z)$ is the stretching potential of a bond and the second equation determines the normalization constant $C(t)$.

Mass conservation of water. According to conservation of incompressible water

$$\partial_t z - \nabla \cdot (\alpha z \nabla P) + jK\left[(P - P_i) - (\Pi - \Pi_i)\right] = 0 \quad \text{in } \mathcal{D}(t), \tag{37}$$

height changes must be balanced by lateral water flow following Darcy's law $\mathbf{v}^{\text{fluid}} = -\alpha\nabla P$ with mobility α (second term) and by water permeation across $\Gamma(t)$ with permeability K (third term).

Mass conservation of osmolytes. Assuming a simple van't Hoff relation, fast equilibration along z, integrating through the thickness and accounting for diffusion and advection with fluid velocity following Darcy's law, it reads

$$\partial_t(z\Pi) - \nabla \cdot (Dz\nabla\Pi) - \nabla \cdot (\alpha z \Pi \nabla P) = 0 \quad \text{in } \mathcal{D}(t). \tag{38}$$

Tangential force balance accounting for variations in the 2D membrane stress and for friction reads

$$\nabla_S\sigma + 2\eta\nabla_S \cdot \mathbf{d} = \mu\mathbf{v} \quad \text{in } \Gamma(t), \tag{39}$$

where η is the membrane viscosity, $\mathbf{d}$ the rate-of-deformation tensor and μ the friction coefficient. For a deforming surface, $\mathbf{d} = (\nabla_S\mathbf{v} + (\nabla_S\mathbf{v})^T)/2 - v_n\mathbf{k}$, where $v_n = (\partial_t z)/j$ is the normal velocity and $\mathbf{k}$ the curvature tensor[8;9]. In our convention, the normal vector to $\Gamma(t)$ points into the vesicle and curvature of a pocket is negative. σ is the Lagrange multiplier field enforcing membrane inextensibility $0 = \nabla_S \cdot \mathbf{v} - v_n H$ where H is the mean curvature.

Normal force balance accounting for hydraulic and Laplace pressures and for bond traction reads

$$0 = P(x, y, t) - P_i + \boldsymbol{\sigma} : \mathbf{k} - j^{-1}T_{\text{bonds}} \quad \text{in } \Gamma(t), \tag{40}$$

where the full 2D stress tensor supported by the membrane is $\boldsymbol{\sigma} = (\sigma - \gamma)\boldsymbol{g} + 2\eta\,\mathbf{d}$ with $\gamma = k_B T c$ the 2D osmotic pressure of bonds, and the bond traction on $\Gamma(t)$ along z is $T_{\text{bonds}} = c\mathcal{V}'(z)$.

Vesicle-scale mechanics and mass conservation. Mechanical force balance in the free-standing part is given by Laplace's law. Force balance at the edge of the patch is given by a Young-Dupré-like equation $k_B T \frac{1}{2\pi r}\int_{\partial\mathcal{D}} c\,d\ell = (1 - \cos\theta)\sigma_v$, where the right-hand-side is the average 2D osmotic pressure of bonds along the edge. Conservation of incompressible enclosed water imposes that the rate of change of volume of the vesicle is balanced by permeation in the free-standing spherical cap and in the adhesion patch, and conservation of inextensible lipids imposes that the total vesicle area remains constant. Finally, conservation of the number of trapped osmolytes N_o imposes that $\Pi_i V_i = (k_B T)N_o$ is constant.

Boundary conditions at the edge of the patch. Because the edge of the patch is not an obstacle for water or osmolyte transport between the external and interstitial media, continuity of hydraulic and osmotic pressures provides boundary data, $P|_{\partial\mathcal{D}} = 0$ (external hydraulic pressure is reference) and $\Pi|_{\partial\mathcal{D}} = \Pi_e$. Similarly, lipids can flow through the edge, and hence $\sigma|_{\partial\mathcal{D}} = \sigma_v$.

Initial conditions. Starting from an equilibrium state for $\Pi_e = \Pi_e^0$ for all unknowns, we suddenly increase external osmotic pressure to $\Pi_e = \Pi_e^0 + \delta\Pi$ at $t = 0$ and self-consistently solve all the equations above over time.

Model parameters. Mass: number of bonds N_b, number of trapped osmolytes N_o, and vesicle surface area. Osmotic pressures: Π_e^0 and shock magnitude $\delta\Pi$. Membrane properties: viscosity η and permeability K. Bonds: stretching potential $\mathcal{V}(z) = \frac{k}{2}(z - z_0)^2$ with k the stiffness and z_0 the resting separation. Interstice: Darcy mobility α, diffusivity D and friction μ. To account for the fact that in detached regions, where $c \sim 0$, bare membrane tension and hydraulic pressure should equilibrate instantly, and hence friction and inverse Darcy mobility should vanish, we assume the relations $\mu(c) = \mu_0 c/c_0$ and $\alpha(c) = \alpha_0 c_0/c$, where μ_0 and α_0 are reference values at the nominal concentration c_0.

Supplementary Note 3: Selection of model parameters

The physical parameters of the theoretical model are the initial vesicle radius R_0, the initial contact angle, the number of bonds N_b (or the nominal bond concentration $c_0 = N_\mathrm{b}/(\pi R_0^2)$), initial osmotic pressure in the medium, the magnitude of the osmotic shock $\delta\Pi$, the bond stiffness k and resting length z_0, the membrane permeability K and viscosity η, and three transport coefficients of the interstice: the osmolyte diffusivity D, the Darcy water mobility α, and the membrane friction μ. Many of the parameters can be taken from the experiments or from available literature, with the exception of D, α, and μ, for which we estimate reasonable ranges based on our experimental conditions and published literature. The parameters used in our simulation in Fig. 2 are reported in Supplementary Table 1. Variations around these parameters are mentioned explicitly.

Membrane separation distance

We estimate the membrane separation distance, z_0 from geometric consideration of molecular sizes. The NAV molecule are 4 nm long and the biotin groups protrude out of the membrane by ~ 0.8 nm, therefore the overall distance between two membranes linked by b-NAV complexes is ~ 5.6 nm[1]. In the DNA systems, we assume that our DNA constructs of 35 base pairs (bp) with a length of 0.38Å\bp[10] behave as rigid rods with a contour length of $\sim$ 12 nm. Therefore the membrane separation distance in the DNA systems is $2 \times 12 + 5.6 \approx 30$ nm, or about 5 times larger than in the b-NAV systems. In our default simulations, we take $z_0 = 7$ nm, whereas in our simulations of the DNA system we take $z_0 = 30$ nm.

Osmotic shock

We consider an initial medium osmolarity of 300 mM, corresponding to $\Pi_\mathrm{e}^0 = 750$ kPa and an osmotic shock of 50 mM, corresponding to $\delta\Pi = 125$ kPa.

Initial contact angle

In the reference simulation of Fig. 2, we consider an initial contact angle of $\theta_0 = 127^\mathrm{o}$ larger than in the experiments of Fig. 1. The change in volume of the vesicle is essentially insensitive to θ_0 and depends only on the osmotic shock, but change in adhesion area is quite sensitive to this angle due to geometry. Spreading is more pronounced as θ_0 decreases. For instance, in Supplementary Video S6 we consider $\theta_0 = 112.6^\mathrm{o}$, leading to a degree of spreading and final contact angle closer to most experiments.

Membrane permeability

We consider a membrane permeability coefficient of $P_\mathrm{f} = 30$ µm/s, in line with previous reports[11;12]. The parameter P_f is related to the permeability coefficient considered here by $K = P_\mathrm{f} V_\mathrm{w}/(RT)$ where $V_\mathrm{w} = 18$ ml/mol is the molar volume of water, R the gas constant and T the absolute temperature, leading to $K = 2 \cdot 10^{-4}$ µm/(s kPa). With the estimate of the time for osmotic equilibration $\tau_\mathrm{osm} = R_0/(K\,\delta\Pi)$, we obtain that vesicles achieve osmotic equilibrium in about 10 min.

Strength of adhesion

As discussed in SI Section 1, we estimate the concentration of bonds in the patch in the range $3 \cdot 10^3$ to $3 \cdot 10^4$ µm^{-2}. For mobile bonds, the adhesion tension γ is the osmotic 2D pressure of these molecules trapped in the adhesion patch and is expressed in a dilute approximation as $\gamma \approx k_\mathrm{B} T c$, where k_B is Boltzmann's constant and T absolute temperature[3;5]. Accordingly, we estimate that in our systems the initial bond tension γ_0 falls between $1.2 \cdot 10^{-2}$ and $2.5 \cdot 10^{-1}$ mN/m. For these values and the typical radius of our vesicles $R_0 \approx 15$ µm, adhesion energy dominates over the bending energy, $\sqrt{\kappa/(\gamma R_0^2)} \ll 1$, where $\kappa \sim 10^{-19}$ J is the bending rigidity of DOPC membranes. Thus, the vesicles are nominally in a strong adhesion limit characterized by

capillary behavior and reduced fluctuations[13;3;5]. In our reference simulation, we consider $\gamma_0 = 2.5 \cdot 10^{-2}$ mN/m.

Membrane friction

The membrane friction coefficient μ in our system results from the combination of several effects, including the resistance to membrane motion by the shearing of the thin and crowded interstitial medium[14] and resistance posed by a forest of obstacles[15;16], here the NAV-bound lipids. Bonds offer resistance to lipid flow because a fraction of them is immobile as measured by FRAP (Fig. S3), and also because mobile bonds will in general move collectively with a different velocity as compared to the lipid effective hydrodynamic velocity. For instance, bonds move away from a nascent and growing blister whereas lipids move towards it. In our model, we do not explicitly account for bond velocity, and hence our approach to modeling friction is simplified.

Typical friction coefficients in supported lipid bilayer systems are in the range 10^{-3} to 10^{-2} kPa s μm^{-1} [14], whereas in cell membranes friction between the plasma membrane and the cytoskeleton is in the order of 2 kPa s μm^{-1} [15]. In our default simulation, we consider an intermediate value of $\mu_0 = 0.1$ kPa s μm^{-1}, and later show that values 10 and 100 times larger lead to very shallow blisters, Fig. 3, inconsistent with our observations. Instead, reducing friction does not change much the results. We thus conclude that our experiments are in a regime in which friction does not play an important role.

To estimate differences in membrane tension $\delta\sigma$ induced by friction, we note that tension gradients first develop between the initial row of pockets and the edge of the adhesion patch, and hence have a characteristic buildup time of $\tau_{\text{mem}} = \ell_{\text{scr}}^2 \mu / \delta\sigma$. On the other hand, we estimate the time for pocket growth as $\tau_{\text{growth}} = \ell_{\text{scr}} / v_{\text{p}} = \ell_{\text{scr}} / (K \delta\Pi)$. Equating these two times and non-dimensionalizing by the osmotic tension scale, we find the dimensionless quantity $\delta\bar{\sigma} = \mu \delta\Pi \sqrt{\alpha z_0 K} / (k_B T c_0)$ characterizing the frictional opposition to pocket growth.

Osmolyte diffusivity

The diffusion coefficient of glucose in water is $D_0 \sim 500$ μm^2, which is thus an upper bound for the diffusion coefficient in the interstitial space, where molecular transport is obstructed by the crowding of linker complexes in a confined interstitial space. The problem of predicting the effective diffusion coefficient in such crowded environments has been extensively studied in the literature[17;18]. Quantitative results depend on specific hypotheses and the degree of crowding but these theories qualitatively agree that diffusivity decreases as the volume fraction of obstacles increases. To obtain estimations, we consider the model of Novak et al.[18].

The glucose molecule has a diameter of 4 nm. In b-NAV systems it diffuses in between b-NAV-b obstacles, that can be modelled as cylinders with a cross-section of 28 nm^2 and a length of $\sim$ 5.6 nm[1]. In the DNA systems, the DNA-b-NAV-b-DNA complexes can be modelled as rods with cross-section of $\sim$ 28 nm^2 and length of $\sim$ 30 nm.

We use $\phi = 1 - (1 - nv)^{v'/v}$ to estimate the inaccessible volume fraction (ϕ) of all linker complexes between two membranes, where n is the number density of the NAV linkers as estimated previously, v is the volume of a single obstacle, and v' is the effective volume of the obstacle, taking into account the size of the osmolyte. From this, we obtain the reduction in the diffusion coefficient (D_{eff}/D_0) using $D_{\text{eff}}/D_0 = (1 - \phi/\phi_c)^{\mu}/(1 - \phi)$, where ϕ_c and μ are fitting parameters determined for a given obstacle aspect ratio[18]. D_{eff}/D_0 is 0.96, 0.8 and 0.14 for b-DOPE densities of 0.2, 1 and 4 mol%, respectively and 0.88 for bDNA-NAV linkers at 4 mol% density. According to these estimations the diffusion coefficient of glucose in the interstitial space should be between 50 and 500 μm^{2}s^{-1}. In our reference simulation, we consider 50 μm^{2}s^{-1}, and perform numerical simulations for 5, 50 and 500 μm^{2}s^{-1} reported in Fig. 3. When simulating the DNA system and following the estimate above, we consider a diffusivity of 440 μm^{2}s^{-1}.

Darcy permeability

An upper bound for the Darcy mobility is given by the Poiseuille limit $h^2/(12\eta_w)$ where $\eta_w \approx 10^{-3}$ Pa s is the viscosity of water[19]. Taking $h = 5.6$ nm, we obtain $\alpha_{\mathrm{Poiseuille}} = 2.6$ μm^2 s^{-1} kPa^{-1}. However, due to molecular crowding, we expect the Darcy mobility to be much smaller. The problem of obtaining the effective Darcy mobility for a viscous fluid moving in a space crowded with obstacles is also one with a rich history[20;21]. Compared to the diffusivity, the Darcy mobility decays to zero faster as crowding increases, in a way that also depends on specific assumptions, making quantitative estimations in this regime very difficult[16]. In our reference simulation, we consider $\alpha = 0.1$ μm^2 s^{-1} kPa^{-1} more than one order of magnitude smaller than the Poiseuille limit, and then consider values 10 times larger and 10 times smaller to study the effect of this parameter in Fig. 3. When simulating the DNA system, we consider a value 10 times larger ($\alpha = 1$ μm^2 s^{-1} kPa^{-1}) than the reference value for the b-NAV system.

Supplementary References

[1] Fenz, S. F., Merkel, R. & Sengupta, K. Diffusion and Intermembrane Distance: Case Study of Avidin and E-Cadherin Mediated Adhesion. *Langmuir* **25**, 1074–1085 (2009).

[2] Amjad, O. A., Mognetti, B. M., Cicuta, P. & Di Michele, L. Membrane Adhesion through Bridging by Multimeric Ligands. *Langmuir* **33**, 1139–1146 (2017).

[3] Bell, G., Dembo, M. & Bongrand, P. Cell adhesion. Competition between nonspecific repulsion and specific bonding. *Biophys. J.* **45**, 1051–64 (1984).

[4] Kaurin, D., Bal, P. K. & Arroyo, M. Peeling dynamics of fluid membranes bridged by molecular bonds: moving or breaking. *Journal of The Royal Society Interface* **19**, 20220183 (2022).

[5] de Gennes, P.-G., Puech, P.-H. & Brochard-Wyart, F. Adhesion Induced by Mobile Stickers: A List of Scenarios. *Langmuir* **19**, 7112–7119 (2003).

[6] de Gennes, P. G. Brownian motion with dry friction. *Journal of Statistical Physics* **119**, 953–962 (2005).

[7] Goychuk, I. & Pöschel, T. Nonequilibrium phase transition to anomalous diffusion and transport in a basic model of nonlinear brownian motion. *Phys. Rev. Lett.* **127**, 110601 (2021).

[8] Arroyo, M. & DeSimone, A. Relaxation dynamics of fluid membranes. *Phys. Rev. E* **79**, 031915 (2009).

[9] Torres-Sánchez, A., Millán, D. & Arroyo, M. Modelling fluid deformable surfaces with an emphasis on biological interfaces. *Journal of Fluid Mechanics* **872**, 218–271 (2019).

[10] Smith, S. B., Cui, Y. & Bustamante, C. Overstretching b-dna: The elastic response of individual double-stranded and single-stranded dna molecules. *Science* **275**, 795–799 (1996).

[11] Olbrich, K., Rawicz, W., Needham, D. & Evans, E. Water permeability and mechanical strength of polyunsaturated lipid bilayers. *Biophysical Journal* **79**, 321–327 (2000).

[12] Bhatia, T., Robinson, T. & Dimova, R. Membrane permeability to water measured by microfluidic trapping of giant vesicles. *Soft matter* **16**, 7359–7369 (2020).

[13] Evans, E. Entropy-driven tension in vesicle membranes and unbinding of adherent vesicles. *Langmuir* **7**, 1900–1908 (1991).

[14] Nissen, J., Gritsch, S., Wiegand, G. & Rädler, J. O. Wetting of phospholipid membranes on hydrophilic surfaces - Concepts towards self-healing membranes. *European Physical Journal B* **10**, 335–344 (1999).

[15] Shi, Z., Graber, Z. T., Baumgart, T., Stone, H. A. & Cohen, A. E. Cell membranes resist flow. *Cell* **175**, 1769–1779.e13 (2018).

[16] Cohen, A. E. & Shi, Z. Do cell membranes flow like honey or jiggle like jello? *BioEssays* **42**, 1900142 (2020).

[17] Dagdug, L., Vazquez, M.-V., Berezhkovskii, A. M., Zitserman, V. Y. & Bezrukov, S. M. Diffusion in the presence of cylindrical obstacles arranged in a square lattice analyzed with generalized fick-jacobs equation. *The Journal of Chemical Physics* **136**, 204106 (2012).

[18] Novak, I. L., Kraikivski, P. & Slepchenko, B. M. Diffusion in cytoplasm: Effects of excluded volume due to internal membranes and cytoskeletal structures. *Biophysical Journal* **97**, 758–767 (2009).

[19] Dasgupta, S., Gupta, K., Zhang, Y., Viasnoff, V. & Prost, J. Physics of lumen growth. *Proceedings of the National Academy of Sciences* **115**, E4751–E4757 (2018).

[20] Tsay, R.-Y. & Weinbaum, S. Viscous flow in a channel with periodic cross-bridging fibres: exact solutions and brinkman approximation. *Journal of Fluid Mechanics* **226**, 125–148 (1991).

[21] Sangani, A. & Acrivos, A. Slow flow past periodic arrays of cylinders with application to heat transfer. *International Journal of Multiphase Flow* **8**, 193–206 (1982).
