# Supplementary material for: Patterning and dynamics of membrane adhesion under hydraulic stress
Source: Nat Commun. 2023 Nov 17;14:7445. doi: 10.1038/s41467-023-43246-7 (PMC10656516; doi:10.1038/s41467-023-43246-7)
Supplement: Supplementary file 3 — Description of Additional Supplementary files [file 41467_2023_43246_MOESM3_ESM.pdf]

Description of Additional Supplementary items

File name: Supplementary Movie 1
Description: Blister formation and coarsening between a vesicle and a SLB, both containing 0.2 mol% biotinylated lipids and subjected to 100 mM hyper-osmotic shock, corresponding to Fig. 1d,e.

File name: Supplementary Movie 2
Description: Simulation of blister formation and evolution, corresponding to Fig. 2b. Model parameters are reported in Supplementary Table S1.

File name: Supplementary Movie 3
Description: Water permeation and interstitial flow in the tightly adhered zone (left) and membrane flow and membrane tension (right) during blister formation and evolution. Model parameters are those of Supplementary Table S1 except the vesicle of contact angle $\theta_0 = 112.6^o$ and system size $R_0 = 2.16$ µm.

File name: Supplementary Movie 4
Description: Blister formation, collapse and adhesion spreading between a vesicle and SLB, containing 6 mol% biotinylated DNA and subjected to 100 mM hyper-osmotic shock. Corresponding to Fig. 4d.

File name: Supplementary Movie 5
Description: Comparison between a simulation with longer linkers representative of the DNA system and a simulation representative of the b-NAV system. DNA linkers are not only longer but also occupy a smaller volume fraction of the interstice, resulting in larger diffusion and Darcy mobility, Supplementary Note 3. The model parameters are those of Supplementary Table S1 except for $R_0 = 15$ µm, $\theta_0 = 90^o$ and in the simulations of the DNA system $z_0 = 30$ nm, $D = 440 \mu m^2 s^{-1}$ and $\alpha_0 = 1\ \mu m^2 s^{-1} kPa^{-1}$.

File name: Supplementary Movie 6
Description: Long simulation reaching over 10 minutes exhibiting blister formation, coarsening and complete adhesion recovery for three vesicles with the same initial contact angle ($\theta_0 = 112.6^o$) and different radii ($R_0 = 1.08$ µm, 2.16 µm, 4.32 µm). The rest of model parameters are those in Sup- plementary Table S1.

File name: Supplementary Movie 7
Description: Mobility of blisters between two adhered vesicles, both containing 0.5 mol% biotinylated lipids and subject to 50 mM hyper-osmotic shock, corresponding to Fig. 4e.

File name: Supplementary Movie 8
Description: Blister budding and shrinking of the adhesion zone between two adhered vesi-cles, both containing 0.5 mol% biotinylated lipids, at later stages following a 100 mM hyper-osmotic shock.
